# Valorization of Green Biomass: Alfalfa Pulp as a Substrate for Oyster Mushroom Cultivation

**DOI:** 10.3390/foods11162519

**Published:** 2022-08-20

**Authors:** Fa Zhou, Mikkel Hansen, Timothy John Hobley, Peter Ruhdal Jensen

**Affiliations:** National Food Institute, Technical University of Denmark, DK-2800 Kgs. Lyngby, Denmark

**Keywords:** green biomass, *Pleurotus ostreatus*, lignocellulose composition, lignocellulolytic enzyme, biodegradation

## Abstract

In this study, the potential of alfalfa pulp as an alternative substrate to wheat straw for the cultivation of oyster mushroom (*Pleurotus ostreatus*) was investigated. The major components associated with different mushroom stages were evaluated, as well as changes in lignocellulolytic enzyme activities in substrates composed of alfalfa pulp, wheat straw or a combination of both. Based on the results, alfalfa pulp was demonstrated to be a better substrate than wheat straw for the production of oyster mushrooms, with a high biological efficiency of 166.3 ± 25.4%. Compared to the cultivation period on commercial straw (31 days), a shorter lifecycle for oyster mushroom was found on alfalfa pulp (24 days), which could help to reduce the risk of contamination during industrial production. Study of the spent substrate as well as the harvested mushrooms revealed that the biological efficiency was related to the higher protein content (17.42%) in the alfalfa pulp compared to wheat straw, as well as greater degradation of cellulose (57.58%) and hemicellulose (56.60%). This was, by and large, due to greater extracellular hydrolytic and oxidative enzyme activity from the mushroom growth in the alfalfa pulp. The quality and safety of the fruiting bodies produced on alfalfa pulp was evaluated, which showed that the protein content was 20.4%, of which 46.3% was essential amino acids, and levels of trace elements and heavy metals were below acceptable limits. Hence, oyster mushroom cultivation using alfalfa pulp provides an alternative method to produce a value-added product, while reducing the biomass wastes in the green protein bio-refinery, and may contribute to sustainable growth in the agricultural industry.

## 1. Introduction

The supply of organic plant-based protein for feed and human consumption, with a suitable amino-acid profile and at a competitive price, is one of the major challenges for agriculture nowadays. Green bio-refineries can produce protein extracts, which contain the required specific amino acids and have the potential to alleviate those alternative protein needs [1]. Since 1969, leaf proteins have been utilized for human consumption as supplements in diets [2]. At the same time, as green biomass bio-refinery concepts become more attractive, it is important that these industries not only pay attention to the production of suitable protein sources, but also to processes that can deal with the huge amount of residues [3]. A recent example of such a green bio-refinery with a high technological readiness level is Biorefine in Denmark [4], and other examples have been recently reviewed by Xiu and Shahbazi [5]. For example, processing of alfalfa with a twin-screw press is an efficient way to extract proteins from the matrix, but, at the same time, it results in 50% dry matter of the raw material being produced as a fibrous residue [6]. Traditionally, most of these solid residues have only been used for animal feeding, and command a low price. However, given the global trend away from animal-based protein sources due to green-house gas emissions, and in order to render the green bio-refinery more sustainable and economically competitive, it is important to look for more sustainable, higher value-added applications. The lignocellulosic residue from alfalfa has a physical structure allowing gas transfer and a suitable carbon/nitrogen ratio that could allow fungal growth. Hoa [7] examined C/N ratios of between 30–50 for growth of *Pleurotus* spp. on various substrates and found that the best performance was at C/N ratios close to 30. One such application could, therefore, be the cultivation of edible, protein-rich mushrooms for human consumption.

Mushrooms are a type of fungi with significant nutritional value and many beneficial properties and currently there are around 2000 edible species distributed around the world [8]. Among the edible mushrooms, oyster mushrooms (*Pleurotus* spp.) represent one of the most common species cultivated and account for more than 16% of the mushroom production industries [9]. In nature, they usually grow on waste materials and colonize dead organic materials, such as dead cottonwood, oak, or maple. During the cultivation period, the mushrooms are able to secret extracellular enzymes, which can degrade the large insoluble components of lignocellulosic materials and provide soluble, low-molecular-weight compounds for growth [10]. These enzymes include different types of peroxidases, such as manganese peroxidase, cellulase and xylanase. In addition to traditional bioconversion of organic wastes into edible protein products, there are other fields where the lignocellulolytic potential of oyster mushrooms may be economically relevant [11]. The residual enzymes in spent mushroom substrate could be a source of enzymes for lignocellulose conversion during second generation ethanol production, or can play a role in remediating soil and industrial wastewater in industry [12].

Generally, the common industrial substrates used for producing edible mushrooms are wheat straw [13], sawdust [14], by-products of cotton [15], and coffee pulp [16]. Important characteristics of these substrates are that they contain lignocellulose, which allows mushroom mycelia development. Though various culture conditions have been tested, the production of oyster mushrooms is usually divided into the following stages: Composting and filling, sterilization, inoculation, incubation, fruiting and harvest [17]. Compared to other species, the cultivation of *P*. *ostreatus* is generally easier, faster, and more cost effective. However, there is often the threat of contamination of the mushroom culture from foreign micro-organisms that affects the mycelia. Sub-optimum growth can result giving low yields of fruit through competition with the mycelia for space and nutrients [18]. Thus, there is an urgent need for an improved mushroom substrate with simple pretreatment, which supports fast mycelium generation and with high fruit productivity.

The present study examines the potential for using alfalfa pulp as a substrate for the production of oyster mushrooms and compares it to the commonly used substrate, wheat straw [13]. We demonstrated that even without the addition of nutrients, the mycelium production was superior to the reference substrate. A comprehensive analysis of the link between *P*. *ostreatus* cultivation and the secretion of enzymes was conducted to evaluate substrate degradation during the different phases of cultivation. Finally, the nutritional and chemical composition of the *P*. *ostreatus* fruiting bodies were examined, including dietary fiber, available sugar, protein, amino-acid profile and chemical composition.

## 2. Materials and Methods

### 2.1. Fungal Strain and Preparation of Alfalfa Pulp

Commercial dried straw and grain spawn of the oyster mushroom strain *Pleurotus ostreatus* were purchased from TagTomat ApS, Copenhagen, Denmark. An amount of 1 kg of fresh alfalfa was directly pressed using a twin screw juicer (Angel Juicer, Busan, Korea), the liquid was saved for green protein production and the resulting pulp was then collected and soaked with deionized water at a 1:1 mass ratio for 2 h before the second pressing. The second pressing was conducted in the same press as used for the first press. The liquid was used for green protein production. The pulp was collected, dried at 80 °C for 24 h to constant weight and then packaged in Ziploc bags and kept at room temperature until used.

### 2.2. Media Preparation and Inoculation

The alfalfa pulp or wheat straw was first ground or chopped with a Kenwood KVL4110W chef machine to a particle size of 0.5–1.0 mm as determined by sieving using a laboratory sifter (Buhler MLUA 230 V). The different dry media (i.e., alfalfa pulp, wheat straw or a mixture of both) were then rewetted with distilled water to give a final moisture content of 80% and sterilized at 121 °C for 15 min. When the substrate had cooled down, 70 g (corresponding to 14 g of dry matter) was placed into a 108 × 81 × 50 mm polypropylene box of 250 mL volume. The media was then inoculated with the spawn of *P*. *ostreatus* (10% of the dry weight of the substrate) by spreading the spawn over the substrate and covering with 0.5–1.0 cm of substrate; then, the prepared boxes were placed into a 300 × 200 mm plastic bag. Each bag was closed with 3 M micropore semi-permeable tape to prevent possible contamination by airborne organisms, while allowing air exchange. The cultures were then placed in an incubator (Aralab climatic chamber, FITOCLIMA 1200 PLH, Sintra, Portugal) where temperature, ventilation, relative humidity and light could be precisely controlled.

### 2.3. Culturing Conditions

Mushroom growth was divided into 4 stages: Fully grown mycelium, phase 1 (P1); primordium, phase 2 (P2); young fruiting bodies, phase 3 (P3), and; mature fruiting bodies, phase 4 (P4). In P1, the temperature and relative humidity were controlled at 24 °C and 85% respectively without lighting, until the substrate was completely colonized; it was assessed by visually observing, through the transparent plastic boxes, the mycelium spreading to the bottom of the substrate. For P2-P4, the culture bags were opened, then the temperature was reduced to 18 °C and the humidity was increased to 90%; additionally, 10% lighting intensity (60 µmoles m^−2^ s^−1^) for 12 h per day was used until the end of P4.

### 2.4. Substrate Conversion

The first flush mushrooms at the end of P4 were harvested by twisting the mushroom at the base of the stem and the mass (both fresh and dry weight) produced by each substrate was determined. To determine the conversion of substrate at each phase of growth, three samples of each substrate were taken randomly then dried to constant weight. The dry weight was compared to that of three samples of uninoculated substrate, which were also dried to constant weight and the biological efficiency (BE) was determined according to Equation (1).

(1) $$\mathrm{BE}=\frac{fresh\;mushroom\;substrate}{dry\;substrate}\times 100\%\;$$

### 2.5. Analysis of Lignocellulose Content in the Substrate

The collected substrate samples were dried at 50 °C for 24 h, then broken up with a Kenwood KVL4110W chef machine and sieved, as described above, into a size range of 0.5–1.0 mm prior to determining the composition and enzyme activities. The Laboratory Analytical Procedures (LAP) established by National Renewable Energy Laboratory (NREL) were used to measure the lignocellulose content [19]. In brief, 0.3 g of biomass was hydrolyzed with 3 mL 72% sulfuric acid for 1 h, then the hydrolyzed biomass was diluted with distilled water to 4% sulfuric acid and autoclaved at 121 °C for 1 h. The hydrolysate was filtered and oven dried to determine the acid insoluble lignin. The filtrate was collected for determining the acid-soluble lignin with the NREL method [17] using a UV-Vis spectrophotometer (Thermo Scientific, GENESYS 10S, Columbus, OH, USA) and monosaccharides by high-performance liquid chromatography. All the monosaccharides were quantified using a Dionex Ultimate 3000 HPLC system equipped with an Aminex HPX-87H column (Bio-Rad, Richmond, CA, USA) and a refractive index detector (Shodex RI-101; Showa Denko K.K., Tokyo, Japan) at 60 °C using 5 mM H_2_SO_4_ as the mobile phase at a flow rate of 0.5 mL min^−^^1^.

### 2.6. Analysis of Enzyme Activity in the Substrate

The substrate was first dried, milled and sieved as described above. Crude enzyme extracts were then obtained by adding 10 mL Na-citrate buffer (pH = 5.0) to 1 g of each sample. The samples were mixed at 4 °C for 24 h and then centrifuged for 20 min at 2500× *g*. The enzyme containing liquid solutions were filtered through 0.22 μm polyethersulfone membrane syringe-filters and used for the different enzyme activity determinations.

The total cellulase activity was determined by the filter paper activity assay according to the method from the standardized NREL Laboratory Analytical Procedure [20]. The value of 2.0 mg of reducing sugar as glucose from 50 mg of filter paper in 60 min has been designated for calculating filter paper cellulase units (FPU) of the enzyme solution. The released reducing sugars were assayed by adding 3 mL of 3,5-dinitrosalicylic acid (DNS) reagent, boiling for 5 min, cooling and diluted with water, then measuring the absorbance at 540 nm.

The Xylanase activity was analyzed according to Bailey et al. [21] using birchwood xylan (1 g L^−^^1^) as substrate. In brief, the released xylose in 5 min at 50 °C was determined by using dinitrosalicylic acid (DNS) reagent at 540 nm. One unit (U) was defined as the amount of enzyme that liberates 1 μmol of xylose equivalents per minute under the assay conditions.

The laccase (Lac) activity was measured by using 2,2′-azino-bis (3-ethylbenzthiazoline-6-sulphonic acid) (ABTS) at a concentration of 0.5 mM in Na-acetate buffer (100 mM, pH = 5.0). The time-dependent oxidation of ABTS was determined by measuring the increase in A_420_ (ε = 36,000 M^−^^1^ cm^−^^1^) [22]. One unit was defined as the amount of enzyme that oxidizes 1 μmol of ABTS per minute.

The lignin peroxidase (LiP) activity was analyzed by measuring the oxidation of 40 mM veratryl alcohol in Na-citrate buffer (100 mM, pH = 4.9) with 0.1 mM H_2_O_2_, spectrophotometrically at 310 nm (ε = 9300 M^−^^1^ cm^−^^1^) according to Haq et al. [23]. One unit was defined as the amount of enzyme that leads to the oxidation of 1 μmol veratryl alcohol per minute.

The manganese peroxidase (MnP) activity was assayed in a mixture of 0.9 mL Na-malonate buffer (100 mM, pH = 5.0) containing 1 mM of manganese ions (Mn^2+^) and 0.1 mL of crude enzyme solution. The reaction was started by addition of 0.1 mM H_2_O_2_ and absorbance was measure at 270 nm. An extinction coefficient of ε = 11.59 mM^−^^1^ cm^−^^1^ was used to calculate the activity and one unit was defined as 1 μmol complex of Mn^3+^-malonate formed per minute [24].

### 2.7. Properties of the Fruiting Bodies

The color of the fresh mushroom pileus was evaluated using a Hunter Lab Miniscan XE colorimeter (Reston, VA, USA). Prior to other analyses, samples of mushroom were frozen dried and crushed with a mortar to give a powder.

The total dietary fiber was analyzed by the Megazyme K-TDFC kit (Megazymes, Bray, Ireland). Briefly, 0.5 g of sample was incubated with α-amylase, protease and amyloglucosidase. Subsequently, the ash and protein content of the residue was determined, and the soluble carbohydrates were measured by the HPLC method described above. The total dietary fiber was then calculated as described in the kit.

The trace metals and heavy metals of the mushroom were analyzed by Hangzhou Yanqu Information Technology Co., Ltd. (Hangzhou, China). Briefly, the dried samples were wet-combusted in HNO_3_ (65%) using a microwave technique (CEN Mars 5) and analyzed by inductively coupled plasma optical emission spectrometry (ICP-OES) [25]. The related estimated daily intake (EDI) and target hazard quotient (THQ) were calculated in Equations (2) and (3) [26].

(2) $$\mathrm{EDI}=\frac{C_{edible\;fungi}\times W_{edible\;fungi\;intake}\;}{B_{average\;weight}}$$

where *C_edible fungi_*, *W_edible fungi intake_*, and *B_average weight_* represent the concentrations of trace metals in the tested fungi (mg kg^−^^1^ DM), the weekly intake of edible fungi (30 g day^−^^1^), and the average body weight (60 kg), respectively [27].

(3) $$\mathrm{THQ}=\frac{EDI\;}{RfD}$$

where oral reference dose (RfD) for Cd, Cr, Cu, Mn, Ni, Fe, Zn, Hg (methyl mercury), and Pb are 0.001, 1.5, 0.04, 0.14, 0.02, 0.7, 0.3, 0.0001, and 0.0036 mg kg^−^^1^ d^−^^1^, respectively [28].

The amino-acid composition was analysed by Hangzhou Yanqu Information Technology Co., Ltd. (Hangzhou, China). HPLC-MS was used, following 0.1 M HCl hydrolysis of the sample and derivatization. The amino acids were identified by comparing retention time and mass spectra of an external standard mixture. The accordingly recommended scoring pattern (RSP) is from the Food and Agriculture Organization (FAO) report [29].

### 2.8. Other Analyses

The total protein content of the samples was estimated by the Dumas (Rapid MAX N exceed cube N/protein analyzer, Elementar Analysensysteme GmbH, Hesse, Germany) using a conversion factor of 6.25 for substrate and 4.38 [23] for oyster mushroom fruit body. The ash content of the samples was determined gravimetrically after incineration at 600 ± 15 °C for 24 h. The extractive content was evaluated by subtracting the content of remaining substances [30].

### 2.9. Statistical Analysis

Each analytical result is reported as the mean value of three replicate sample measurements, except where stated. Standard deviations and statistical differences were analyzed using Origin 2021. Differences between the means of samples were analysed by Fisher’s least significant difference (LSD) test at a probability of 0.05.

## 3. Results and Discussion

### 3.1. Mushroom Growth on Different Substrates

The objective of this study was to assess the potential of alfalfa pulp for the production of mushrooms. In order to do this, a comparison was made with growth on a conventional mushroom growth medium (straw) and a mixture of the two substrates, i.e., straw and alfalfa pulp. The composition of the three substrates was determined and the results showed that the alfalfa pulp had lower concentrations of cellulose, hemicellulose, soluble and insoluble lignin, total fiber and ash, but higher concentrations of protein, nitrogen and extractives than wheat straw (Table S1 in Supplementary Materials). In all cultivations, the same inoculum particle size (0.5–1.0 mm) and spawn inoculation level (10%) were used, without any nutritional supplements. In general, spawn running time on different substrates with different cultivation methods should allow for harvesting of mature fruiting bodies 5 days after emergence of the pins and the total cultivation period can normally be expected to be approximately 30–35 days [31].

The results in Table 1 show that the alfalfa pulp performed very well as a growth substrate and shortened the growth time at each phase when compared to straw and to a mixture of alfalfa pulp and straw substrate. Button-shaped mushroom bodies appeared in 22 days (P3), and two days after that the first mature fruit could be harvested (P4) when alfalfa pulp was used. The longest time for each phase was seen when straw only was used and the addition of alfalfa pulp to the straw was able to shorten these times (Table 1). The biological efficiency (BE) value (166.30 ± 25.44%) of *P*. *ostreatus* when grown on alfalfa pulp was much higher than the other two substrates and exceeded 100%. This could indicate that it is an ideal substrate. Furthermore, when compared with the literature for other substrates, the BE value of alfalfa pulp (166.30 ± 25.44%) is higher than wheat straw (50.2%), coffee pulp (86.5%) [16], and faba bean hulls (109%) [25]. For example, Vieira and de Andrade [32] investigated different substrates for commercial *P*. *ostreatus* growth in a production-scale setting and found BE values of ca. 66% when using wheat straw without nutritional supplementation compared to ca. 80% using sugar cane straw without supplementation. The BE of the wheat straw substrate used in the current experiment with the same species was 22.72 ± 0.14%, which is lower than that reported by Vieira and de Andrade [32] and when compared with 54.2 ± 11.8% reported by Salmones et al. [16]. The difference could possibly be due to the small-scale experiment in this study, with initial dry substrate weight around 14 g per test, in contrast to the scale used by Salmones et al. [16], which was 200 g dry substrate. However, it is more likely that a difference in conditions is responsible for the difference in BE, since the yield can be improved by optimizing the culture conditions [17]. If so, then even higher BE values may be obtained in commercial production settings when using alfalfa pulp. Furthermore, the BE value of the straw could be raised dramatically from 22.72 ± 0.14% to 101.85 ± 16.88%, when it was mixed with alfalfa pulp. This suggests that the alfalfa pulp could also be used as an additive in a well-functioning mushroom production system.

**Figure 1:**
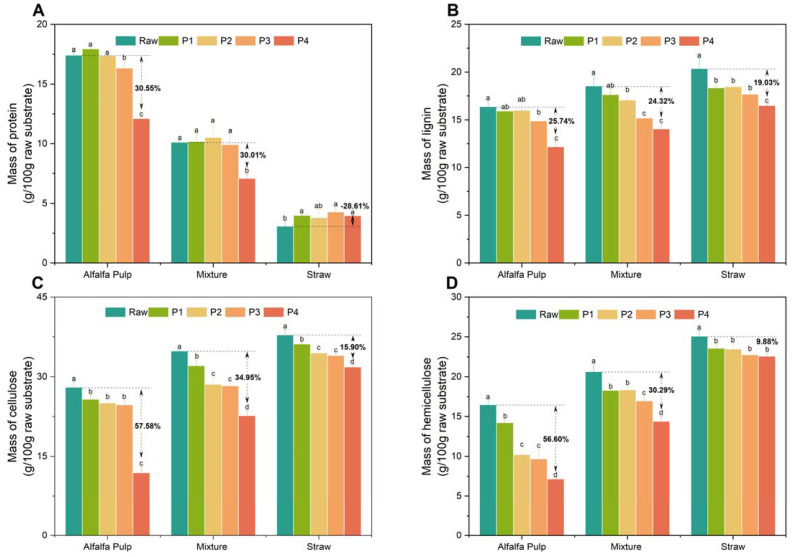
Major components of the mushroom substrates at different cultivation phases. Mass of: (**A**) protein; (**B**) lignin; (**C**) cellulose; (**D**) hemicellulose in g 100 g^−^^1^ raw substrate. The results are averages and standard deviations of 3 biological replicates. P1–P4 designate the cultivation stage. P1, fully grown mycelium; P2, primordium; P3, young fruiting bodies; P4, mature fruiting bodies. The letters a–d indicate if there is a significant difference (*p* ≤ 0.05) between different stages in the substrate determined using Fisher’s least significant difference.

To understand why different substrates have different bioconversion efficiencies, a detailed analysis of the mushroom substrates composition at different stages was performed, see Figure 1. The results show that mushroom formation was related to the degradation of components in the substrate. Furthermore, the type of substrate has a major influence on the level of degradation, which is also reflected in the BE value (Table 1). The results in Figure 1 show that alfalfa pulp has more protein and less lignin, cellulose and hemicellulose than straw, or the mixture of alfalfa and straw. Furthermore, it can be seen that for almost all substrates, the amounts of protein, lignin, cellulose and hemicellulose decrease significantly during mushroom growth from the P1 to P4 phase. The only exception being for the straw substrate. The more than 3-fold higher protein content in the alfalfa pulp (Figure 1A) compared to the straw may indicate that straw lacks sufficient organic nitrogen content, thus leading to the best cultivation performance on the alfalfa pulp. It is known that nitrogen is also a major factor that affects enzyme secretion (cellulases, hemicellulases, and laccases), which is important in the degradation of cellulose, hemicelluloses and lignin, respectively [33]. Although it is known that nitrogen excess can negatively affect the degradation of lignin [33], no evidence of that was seen when comparing the alfalfa pulp and straw degradation (Figure 1A). Supplementation of the straw with alfalfa pulp was seen to increase the productivity and biological efficacy of the oyster mushroom (Table 1), which is consistent with the increase in protein in the mixture, compared to the straw only (Figure 1A).

A positive correlation was also observed between the BE value (Table 1) and cellulose and hemicellulose degradation in all spent substrates (Figure 1C,D). The degradation of cellulose was 57.58%, 34.95% and 15.90% in the alfalfa, mixture and straw, respectively. According to Owaid et al. [34], some mixed substrates were made to overcome the low bioconversion and render them more popular and acceptable in the mushroom cultivation industry than using straw alone. It was of interest in the current work that the greatest degradation of cellulose happened in the final stage (P4) when using alfalfa pulp and the mixture. Normally, the lignin, cellulose, and hemicellulose of the substrate will be utilized evenly during mushroom cultivation stages [35]. The differences in this study could possibly be due to changes in the levels of hydrolytic enzymes secreted during the different growth phases, which cause the simultaneous or selective degradation of cellulose and hemicelluloses along with lignin [36]. Evidence of selective degradation of hemicellulose during different growth phases was observed in Figure 1D for alfalfa pulp, where there the majority of the decrease was seen from the P1-P2, in contrast to the mixture and straw only. Overall, more cellulose and hemicellulose was degraded compared to lignin for the alfalfa pulp compared to the straw. In contrast, Angel et al. [37] reported that *Pleurotus* spp. are efficient lignin degraders, and are able to remove more lignin selectively from non-woody lignocellulosic materials. It is speculated that the low lignin degradation of the non-woody alfalfa pulp can be attributed to the mechanical pressing pretreatment of the alfalfa. Here, the amorphous and crystalline cellulose matrix in the biomass residues might be disrupted, which may then positively affect the bioconversion [38].

**Figure 2:**
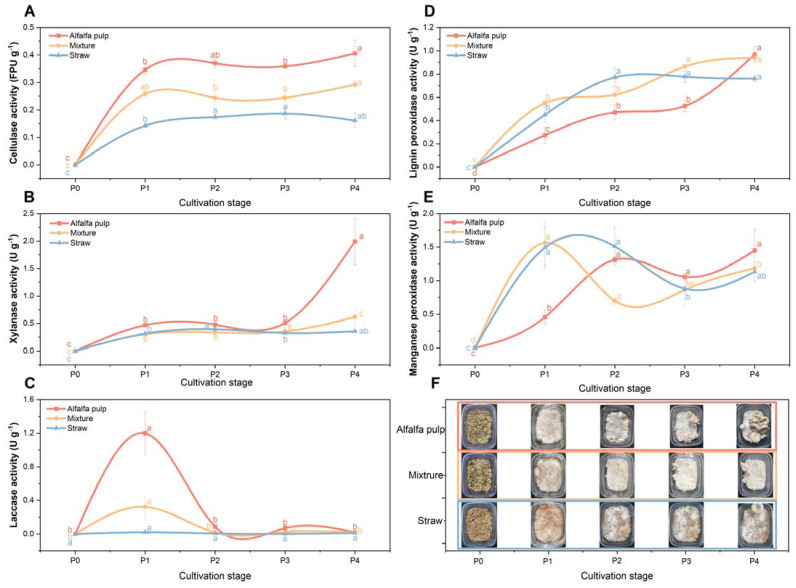
Enzyme activity during growth on substrates. (**A**) cellullases activity; (**B**) xylanases activity; (**C**) laccase activity; (**D**) lignin peroxidase activity; (**E**) manganese peroxidase activity; (**F**) morphology of the different cultivation stages of *P*. *ostreatus*. P1–P4 designate the cultivation stage. P1, fully grown mycelium; P2, primordium; P3, young fruiting bodies; P4, mature fruiting bodies. The results show averages and standard deviations of three biological replicates. The letters a–c indicate if there is a significant difference (*p* ≤ 0.05) between different phases, determined using Fisher’s least significant difference.

*P*. *ostreatus*, as the traditional white rot fungi, uses extracellular enzymes to form a ligninolytic and a hydrolytic system to degrade lignocellulose. It can be expected that the selective degradation of components in the alfalfa pulp during the different stages of mushroom cultivation would be due to three main enzymes: cellulases, xylanase and the ligninolytic peroxidase. The activity of these enzymes during *P*. *ostreatus* growth on all test substrates was thereby examined, and the results are shown in Figure 2. The related morphology of four growth phases of *P*. *ostreatus* on three substrates are in Figure 2F.

Cellulase activity was highest on alfalfa (0.40 ± 0.05 U g^−^^1^), next highest on the mixture and the least activity was observed on the straw. The activities increased most until the P1 stage and were then, more or less, constant until mushroom harvesting for all substrates. This is consistent with the degradation pattern seen in Figure 1C, where continuous cellulose degradation occurred during the cultivation process. It is also consistent with the highest BE values seen on the alfalfa pulp in Table 1. Cellulases have different specificities to hydrolyse the β-1,4-glycosidic linkages and convert the polysaccharides to oligosaccharides for fungi growth and metabolism [39]. The values found here for cellulase are similar to those found in a previous study [40], which recovered the same amount of enzyme in spent mushroom composts as a product.

Unlike cellulase activity, xylanase activity was low for the first three phases then increased dramatically at P4 (2.00 ± 0.42 U g^−^^1^) in alfalfa pulp and to a lesser, though significant amount for the mixture (0.62 ± 0.14 U g^−^^1^) (Figure 2B). The trend observed here is consistent with other studies, which have shown that xylanase activity on mushroom substrate increased over time and was associated with the fruiting body formation [24]. Because xylan is the major constituent in hemicellulose, this result is consistent with the hemicellulose loss (Figure 1D) seen for the alfalfa pulp and the mixture with straw. It is known that nitrogen sources have a dramatic effect on the production of xylanase [41]. Although straw has very high hemicellulose levels (25.06 ± 0.74%), the lack of nitrogen compared to the alfalfa pulp and the mixture appears to have limited xylanase production.

Lignin degradation is a complex process. Previous studies indicated that the lignin peroxidase is active in the primordium and the fruiting body formation stages. Here there is oxidation of the non-phenolic units of lignin, cleavage the C_α_-C_β_ bond in lignin molecules, and opening of the ring of the aromatic skeleton [42]. Manganese peroxidase oxidizes a bound Mn^2+^ to Mn^3+^ in the presence of hydrogen peroxide generating an intermediate redox couple Mn^2+^/Mn^3+^. The Mn^3+^ complex can diffuse into the lignified cell wall, where it oxidizes phenolic or nonphenolic lignin components [43]. However, laccase is strongly inhibited by H_2_O_2_ [41]. It can, therefore, be expected that lignin peroxidase and manganese peroxidase activity in the substrate would increase during cultivation, but that laccase production would show a different pattern due to inhibition by H_2_O_2_.

When the results in Figure 2C, D are inspected, trends are observed that are consistent to what is expected from the literature above. There was a dramatic spike in laccase activity (Figure 2C) at the P1 phase for the alfalfa substrate and to a lesser extent for the mixed substrate, after which it declined to zero by phase P2. Ruiz-Rodríguez et al. [44] also found that laccase activity reached a maximum (1.2–2.1 U g^−^^1^) after around 10–15 days of cultivation with six *Pleurotus* spp. strains on wheat straw, which was then followed by a significant decrease in activity [44].

In contrast, the major ligninolytic enzymes lignin peroxidase (Figure 2D) and manganese peroxidase (Figure 2E) showed a trend of increasing during cultivation. The highest values for lignin peroxidase were seen in the alfalfa pulp (1.20 ± 0.26 U g^−^^1^) and occurred when the fruiting bodies had formed, although the activities of this enzyme were generally lower than for the straw and the mixed substrate in the other growth phases (Figure 2). The lignin peroxidase activity gradually increased and reached a first peak at different stages for the different substrates. Maximum activity (0.77 ± 0.08 U g^−^^1^) was observed at P2 for straw and with no significant increase during the rest of cultivation (*p* ≤ 0.05). During growth on alfalfa pulp and the mixture, the point of this peak moved to P4 with 0.97 ± 0.06 U g^−^^1^ and P3 with 0.86 ± 0.02 U g^−^^1^, respectively. The manganese peroxidase enzyme activity monitored in this study oscillated. A peak in activity was seen during P1 for the straw and mixture and at P2 for the alfalfa pulp. After these peaks, activity declined, then increased again until the fruiting body was harvested (Figure 2E) at P4. This is consistent with a report by Velázquez-Cedeño et al. [45], who observed manganese peroxidases appeared in coffee pulp until the end of the incubation period.

In lignocellulosic degrading systems, various enzymes act together to produce sugars that can be easily assimilated by the mushroom’s mycelium. Although alfalfa pulp has a low lignocellulosic profile compared to other substrates utilized in this study, higher enzymatic activity observed in the early stage would partially explain the successful cultivation results obtained. Thus, in principle, a media which has a better capacity for *P*. *ostreatus* colonizing, synthesizing and secreting ligninolytic enzymes could produce higher fruiting bodies yields [44].

### 3.2. The Chemical Composition and Nutritional Value of Harvested Mushrooms

The above results indicated that 14.10 g of fresh mushrooms were produced from 14.56 g of dry alfalfa pulp and that this is an effective process to upcycle the pulp. However, it is also important that the mushrooms produced have the right quality, as described by their nutritional and chemical composition. It has been reported that the nutritional composition is highly variable when using different cultivation substrates [13]. These properties were therefore analysed and the results are shown in Table 2.

The general trend of the results in Table 2 is that the mushrooms produced had similar, if not better, nutritional properties compared to what has been reported in the literature. For example, the total protein content on a dry weight basis was 20.36 ± 2.90% (Table 2). Koutrotsios et al., reported protein content of mushrooms grown on wheat straw and date-palm tree leaves of 14.64 ± 1.38% and 16.13 ± 1.22%, respectively [46]. The total dietary fiber, which is associated with anti-carcinogenic properties and immune regulatory functions [47], of the alfalfa pulp grown mushrooms was 28.24 ± 0.01% (Table 2), which is within the range reported for edible *P*. *ostreatus* (10.60–57.00%) [13]. Furthermore, the available carbohydrates consisted mainly of trehalose (21.00 ± 1.15%) and glucose (11.40 ± 0.11%), which were at similar levels to those previously found in other studies [48].

Lighting is an extrinsic factor affecting mushroom cultivation, which can change the color of the cap (pileus) of the oyster mushroom from bright white to dark, through releasing oxidized phenols by phenoloxidase and forming melanoidins. In this study, 12 h d^−1^ of lighting was used during the final cultivation stage (P3-P4) and the ΔL (lightness), Δa (redness), Δb (yellowness) of the resulting mushrooms were 73.00 ± 2.40, −1.34 ± 0.52, and 16.90 ± 0.82, respectively. These values indicated higher brightness and lower redness than other researchers measured [49], however, there is no relevant standard for this appearance quality.

The mineral composition of fruiting bodies of *P*. *ostreatus* produced from alfalfa pulp and the potential risk due to heavy metals was investigated and the results are shown in Table 3. The major minerals in the tested mushroom were found to be K (11,176 ± 1858 mg kg^−^^1^ DM), P (9843 ± 1391 mg kg^−^^1^ DM), and Ca (2785 ± 1633 mg kg^−^^1^ DM). This is consistent with other reports that pointed out that from a nutritional point of view, *P*. *ostreatus* has high levels of potassium and phosphorous, which are beneficial for control of blood pressure [12]. The mushrooms provide higher Ca values than almonds (2640 mg kg^−^^1^) and kale (1500 mg kg^−^^1^) [35]. Oyster mushrooms are well-known for accumulating heavy metals, the concentration of which is affected by the growth substrates. Substrates high in a particular mineral produce mushrooms relatively high in the content of that mineral [50]. The results in Table 3 show that the estimated daily intake of Pb, Cd, Ni, Cr, and Hg from eating the tested mushrooms was lower than the provisional tolerable daily intake. Through further analysis of THQ, it could be seen that Cu, Mn, Zn, Fe, Pb, Cd, Ni, Cr, and Hg were all within the safe range (THQ ≤ 1) [36]. The value of total THQ (0.75) was lower than the edible mushrooms standard in Zambia (2.79), Serbia (2.30), Slovakia (1.65) Romania (1.59), China (1.39), Poland (0.98), Italy (0.89), and Greece (0.77), but higher than in Ukraine (0. 58), Bulgaria (0.45) and Korea (0.20) for adults [28].

Nutritionally speaking, the protein quality of *P*. *ostreatus* is one of its major strengths because it has a high content of all the essential amino acids and excellent protein digestibility [51]. After the analysis of seventeen amino-acids in *P*. *ostreatus* produced on the alfalfa pulp, it was found that the total amino acid content (213.52 ± 5.35 mg g^−1^ DM; Table 4) was equivalent to the protein content measured (20.36 ± 2.90%; Table 2). The dominant amino acids in the mushrooms from alfalfa pulp were Asp and Glu (Table 4), which accounted for 19.52 ± 0.06 and 29.01 ± 0.62 mg g^−1^, respectively, of the dry mushroom weight and are similar to the values reported previously in the literature [52]. In addition, the harvested mushroom meets the nutritional requirements of all essential amino acids for adults (Lys, Leu, Val, Ile, AAA: aromatic amino acids (Phe + Tyr)) and it was noteworthy that their composition meets the recommended scoring pattern (RSP) for adults. Threonine, SAA (sulphur amino acids: (Cys + Met)), His, and Lys are more than twice the recommended levels, and the aromatic amino acids are more than four times. Interestingly, the total essential amino acid content measured here is higher for *P*. *ostreatus* grown on perilla stalks (25.38%) and cotton-seed hull (27.69%) [15]. The oyster mushrooms grown on alfalfa pulp have an excellent nutritional profile and no negative characteristics as measured here. It can be speculated that the high quality proteins known to be present in alfalfa [53], may positively affect the amino-acid composition of *P*. *ostreatus* fruiting body.

## 4. Conclusions

This study has demonstrated that alfalfa pulp had better performance than straw for producing oyster mushrooms, without the need for further nutritional additions. Compared to the cultivation period of 31 days on straw, alfalfa pulp produced the fruiting bodies within only 24 days, which is important for avoiding contamination. The reasons for higher biological efficiency achieved on alfalfa pulp are concluded to be a combination of the high protein content in the substrate compared to wheat straw, and higher production of enzymes needed for the breakdown of the lignocellulosic and hemicellulosic structure. Moreover, the *P*. *ostreatus* produced from alfalfa pulp have safe trace-metals concentration ranges, as well as excellent protein content and essential amino acids profile. Using alfalfa pulp for oyster mushroom cultivation is concluded to be a promising alternative application for this byproduct after protein extraction. Alfalfa pulp is recommended as a substitute for wheat straw given the faster production of fruiting bodies and superior biological efficiency.

## Figures and Tables

**Table 1:** Cultivation parameters for *P*. *ostreatus* mushrooms produced on three substrates: Alfalfa pulp, mixture of straw and alfalfa pulp and straw only. Results are given as mean ± standard deviation, *n* = 3 biological replicates. P1–P4 designate the cultivation stage: P1, fully grown mycelium; P2, primordium; P3, young fruiting bodies; P4, mature fruiting bodies.

		Weight (g)	Cultivation Stages and Number of Days Needed				Substrate Dry Matter Loss (%)	Biological Efficiency (BE) (%)
			P1	P2	P3	P4		
Alfalfa pulp	Initial dry substrate	14.56 ± 0.09	6.67 ± 0.29	18.67 ± 1.53	22.33 ± 1.15	24.25 ± 0.56	41.51 ± 3.01	166.30 ± 25.44
	Spent dry substrate	8.51 ± 0.39						
	Fresh mushroom	14.10 ± 2.17						
Mixture (50:50)	Initial dry substrate	14.48 ± 0.35	7.33 ± 0.57	24.00 ± 1.73	26.50 ± 0.70	28.67 ± 2.08	30.29 ±5.20	101.85 ± 16.88
	Spent dry substrate	10.01 ± 0.68						
	Fresh mushroom	10.21 ± 1.39						
Straw	Initial dry substrate	14.70 ± 0.03	7.80 ± 0.80	26.67 ± 1.15	28.00 ± 1.00	31.33 ± 1.53	8.20 ± 2.42	22.72 ± 0.14
	Spent dry substrate	13.49 ± 0.33						
	Fresh mushroom	3.07 ± 0.05						

**Table 2:** The properties of *P*. *ostreatus* mushroom harvested from alfalfa pulp. Results are given as mean ± standard deviation, *n* = 3 biological replicates. DM = dry matter.

Parameters Examined		Values Measured
Moisture (g/100 fresh weight)		83.13 ± 0.01
Ash (g/100 DM)		1.84 ± 0.16
Protein (g/100 DM)		20.36 ± 2.90
Total dietary fiber (g/100 DM)		28.24 ± 0.01
Available carbohydrate (g/100 DM)	D-Trehalose	21.00 ± 1.15
	D-glucose	11.40 ± 0.11
	D-xylose	1.03 ± 0.04
Color	ΔL	73.00 ± 2.40
	Δa	−1.34 ± 0.52
	Δb	16.90 ± 0.82

**Table 3:** The concentration of elements (mg kg^−^^1^ DM) measured in the *P*. *ostreatus* mushrooms harvested from alfalfa pulp, the resultant estimated daily intake (EDI) and the target hazard quotient (THQ). Provisional tolerable daily intake (PTDI) for heavy metals appear in parentheses. Results are given as mean ± standard deviation, *n* = 3 biological replicates.

Trace Elements	Fruiting Body (mg kg^−1^ DM)	EDI (µg kg^−1^ Body Weight^−1^ Day^−1^)	THQ
K	11,176.51 ± 1858.37	942.74 ± 156.75	-
P	9843.66 ± 1391.04	830.31 ± 117.33	-
Ca	2785.73 ± 1633.49	234.98 ± 137.78	-
Mg	1023.90 ± 141.71	86.37 ± 11.95	-
Na	256.67 ± 56.95	21.65 ± 4.80	-
Fe	135.35 ± 63.54	11.42 ± 5.36	0.03
Zn	71.52 ± 1.73	6.03 ± 0.15	0.14
Mn	25.82 ± 14.07	2.18 ± 1.19	0.17
Cu	12.02 ± 0.54	1.01 ± 0.05	0.03
Heavy Metals			
Pb	10.01 ± 0.62	0.84 ± 0.05 (3.57)	0.13
Cr	1.42 ± 0.77	0.12 ± 0.06 (100)	0.23
Cd	0.09 ± 0.01	8.20 × 10^−3^ ± 5.89 × 10^−4^ (1)	4 × 10^−3^
Ni	0.85 ± 0.42	0.72 ± 0.04 (5)	6 × 10^−3^
Hg	0.02 ± 0.02	1.73 × 10^−3^ ± 1.27 × 10^−3^ (0.71)	0.02

**Table 4:** The amino acid concentration (mg g^−1^ DM) of harvested *P*. *ostreatus* mushrooms. Results are given as mean ± standard deviation, *n* = 3 biological replicates. RSP is the recommended scoring pattern.

Amino Acid	This Study (mg g^−1^ DM)	RSP (mg g^−1^ DM)
Aspartic acid (Asp)	19.52 ± 0.06	-
Serine (Ser)	9.81± 0.23	-
Glutamate (Gln)	29.05 ± 0.62	-
Glycine (Gly)	9.30 ± 0.28	-
Alanine (Ala)	12.18 ± 0.87	-
Arginine (Arg)	11.29 ± 0.05	-
Proline (Pro)	13.65 ± 0.50	-
Threonine (Thr)	10.17 ± 0.22	5.09
Valine (Val)	12.50 ± 0.37	8.14
Cystine (Cys)	0.77 ± 0.09	4.68
Methionine (Met)	7.36 ± 0.03	4.68
Isoleucine (Ile)	11.25 ± 0.27	6.10
Leucine (Leu)	14.96 ± 0.58	12.41
Tyrosine (Tyr)	9.19 ± 0.25	5.09
Phenylalanine (Phe)	16.61 ± 0.04	5.09
Histidine (His)	7.73 ± 0.28	3.23
Lysine (Lys)	18.16 ± 0.99	9.77
Total AAs content	213.52 ± 5.35	-
Total essential AAs content	98.75 ± 2.78	-
Essential AAs/FAAs (%)	46.25 ± 0.01	-

## Data Availability

The date are available from the corresponding author.

## Supplementary Materials

The following supporting information can be downloaded at: https://www.mdpi.com/article/10.3390/foods11162519/s1, Table S1. Chemical composition of three test substrates. The values are averages and standard deviations of 3 biological replicates. DM = dry matter.
