# Supplementary material for: Valorization of Green Biomass: Alfalfa Pulp as a Substrate for Oyster Mushroom Cultivation
Source: Foods. 2022 Aug 20;11(16):2519. doi: 10.3390/foods11162519 (PMC9407111; doi:10.3390/foods11162519)
Supplement: Supplementary file 1 [file foods-11-02519-s001.zip › foods-1863247-supplementary.pdf]

Table S1. Chemical composition of three test substrates. The values are averages and standard deviations of 3 biological replicates. DM= dry matter.

Compounds (g/100g DM)	Alfalfa pulp	Mixture (50:50)	Straw
Ash	1.83 ± 0.23 %	2.30 ± 0.10 %	1.89 ± 0.12 %
Total Fiber	60.88 ± 1.34 %	74.05 ± 0.99 %	83.29 ± 1.76 %
Cellulose	28.01 ± 1.14 %	34.86 ± 0.03 %	37.86 ± 1.15 %
Hemicellulose	16.48 ± 0.70 %	20.64 ± 1.02 %	25.07 ± 0.75 %
Acid soluble lignin	1.40 ± 0.01 %	1.32 ± 0.02 %	1.75 ± 0.01 %
Acid insoluble lignin	16.39 ± 0.64 %	18.55 ± 1.10 %	20.37 ± 1.11 %
Protein	17.42 ± 0.24 %	10.12 ± 0.15 %	3.09 ± 0.04 %
Nitrogen	2.79 ± 0.01 %	1.62 ± 0.01 %	0.45 ± 0.02 %
Extractives	20.65 ± 0.01 %	13.05 ± 0.84 %	12.73 ± 0.81 %
